# Reliability and validity of using the Lokomat to assess lower limb joint position sense in people with incomplete spinal cord injury

**DOI:** 10.1186/1743-0003-11-167

**Published:** 2014-12-16

**Authors:** Antoinette Domingo, Tania Lam

**Affiliations:** School of Kinesiology, University of British Columbia, Vancouver, BC Canada; International Collaboration on Repair Discoveries (ICORD), Vancouver, BC Canada

## Abstract

**Background:**

Proprioceptive sense (knowing where the limbs are in space) is critical for motor control during posture and walking, and is often compromised after spinal cord injury (SCI). The purpose of this study was to assess the reliability and validity of using the Lokomat, a robotic exoskeleton used for gait rehabilitation, to quantitatively measure static position sense of the legs in persons with incomplete SCI.

**Methods:**

We used the Lokomat and custom software to assess static position sense in 23 able-bodied (AB) subjects and 23 persons with incomplete SCI (American Spinal Injury Association Impairment Scale level B, C or D). The subject’s leg was placed into a target position (joint angle) at either the hip or knee and asked to memorize that position. The Lokomat then moved the test joint to a “distractor” position. The subject then used a joystick controller to bring the joint back into the memorized target position. The final joint angle was compared to the target angle and the absolute difference was recorded as an error. All movements were passive. Known-groups validity was determined by the ability of the measure to discriminate between able-bodied and SCI subjects. To evaluate test-retest reliability, subjects were tested twice and intra-class correlation coefficients comparing errors from the two sessions were calculated. We also performed a traditional clinical test of proprioception in subjects with SCI and compared these scores to the robotic assessment.

**Results:**

The robot-based assessment test was reliable at the hip and knee in persons with SCI (P ≤ 0.001). Hip and knee angle errors in subjects with SCI were significantly greater (P ≤ 0.001) and more variable (P < 0.0001) than in AB subjects. Error scores were significantly correlated to clinical measure of joint position sense (r ≥ 0.507, P ≤ 0.013).

**Conclusions:**

This study shows that the Lokomat may be used as a reliable and valid clinical measurement tool for assessing joint position sense in persons with incomplete SCI. Quantitative assessments of proprioceptive deficits after neurological injury will help in understanding its role in the recovery of skilled walking and in the development of interventions to aid in the return to safe community ambulation.

**Electronic supplementary material:**

The online version of this article (doi:10.1186/1743-0003-11-167) contains supplementary material, which is available to authorized users.

## Background

Spinal cord injury (SCI) often results in complete or partial paralysis, affecting the ability to walk and participate in physical activity. Because of this diminished mobility, people with SCI are at high risk of secondary complications such as compromised cardiovascular health, pressure sores, and osteoporosis [1, 2]. Therapeutic interventions that can improve walking ability are important because they can help to reduce these secondary complications and increase participation and quality of life.

Strategies to improve ambulation in people with SCI have largely focused on enhancing motor output [3]. Intensive, task-specific gait retraining strategies that provide repeated practice of walking movements have been shown to improve walking function [4–6]. It is thought that the sensory information provided by the repeated practice of movements promotes neural plasticity through use-dependent mechanisms [4, 7].

One key sensory modality critical for the control of coordinated movements, including walking, is proprioceptive sense - the sense of position and movement (kinesthesia) [8]. This is perhaps best illustrated by a case report of a person who had lost all proprioceptive sensation below the neck [9]. This person had to compensate for compromised balance and stability by visually monitoring his steps and using a larger base of support [9]. In cases of people with pyridoxine (vitamin B6) toxicity, which damages large-diameter afferents, there have been reports of ataxic gait, demonstrating the important role of proprioception in inter-joint coordination during walking [10]. Also, impairments in obstacle crossing in people with peripheral neuropathy secondary to diabetes have been associated with impaired proprioception [11]. Given that a SCI could damage ascending proprioceptive tracts, it seems vital to understand how proprioceptive deficits in people with SCI impact functional ambulation, especially when performing skilled locomotor tasks such as walking over uneven terrain, obstacle crossing or stair negotiation.

Clinical assessments of proprioceptive sense used by clinicians are not quantitative and lack sensitivity [12]. For example, one clinical measure of joint position sense involves the clinician grossly moving a limb and asking the patient to simply indicate the direction that the limb was moved [13]. Another measure of proprioception involves imitating a presented movement but quantification of the response is usually only estimated [14]. In addition, when performing these manual test, the velocity of movement, points of contact, and force of contact applied to the individual can vary, affecting the results of the examination. The administration of these manual tests is very difficult to standardize and quantify.

Several groups have developed methods to quantitatively measure proprioception in the upper extremities of able-bodied subjects [15–19]. In addition, tools have been developed to quantitatively measure joint position sense in the upper extremity in persons with stroke [20–22] and hemiplegic cerebral palsy [18]. There are a number of studies that quantitatively measure kinesthesia in the lower limb [23–26] and proprioception related to joint dysfunction [27–30]. However, there are no tools that are suitable for quantitative testing of lower limb proprioception in people with neurological injury. A reliable and precise method to measure proprioceptive sense in the lower limbs, especially one that could be used for neurological populations, is needed. Precise clinical assessments of sensory function are necessary to evaluate the effectiveness of treatments and understand the role of proprioceptive sense in locomotor recovery. This is an essential first step that precedes the development of treatments to improve sensory function and ultimately maximize skilled walking and community ambulation.

Thus, the purpose of our study was to test the reliability and validity of a new quantitative assessment tool of lower limb joint position sense using the Lokomat (Hocoma AG, Volketswil, Switzerland), a robotic gait rehabilitation device. We hypothesized that the Lokomat-based assessment of proprioceptive sense would be a reliable and valid method of measuring conscious proprioception in persons with incomplete spinal cord injury (iSCI).

## Methods

### Subjects

Individuals with iSCI were recruited to participate. Participants had to meet the following inclusion criteria: 1) at least 6 months post injury; 2) were in stable medical condition; 3) no history of musculoskeletal disease; 4) no cardiovascular condition where exercise was contraindicated; 5) weight was less than 300 lbs and height was less than 6’1” due to the capacity limits of the robotic exoskeleton; 6) 19 years of age or older; 7) able to follow directions so they could complete the experiment.

Able-bodied participants were included if they: 1) had no neurological, cardiovascular, or musculoskeletal injuries interfering with their ability walk 2) weight was less than 300 lbs and height was less than 6’1” due to the capacity limits of the robotic exoskeleton; 3) 19 years of age or older.

Subjects participated in this study with informed, written consent. Experimental procedures were approved by the Research Ethics Board at the University of British Columbia and were conducted in accordance with the Declaration of Helsinki.

### Robotic assessment

**Figure 1:**
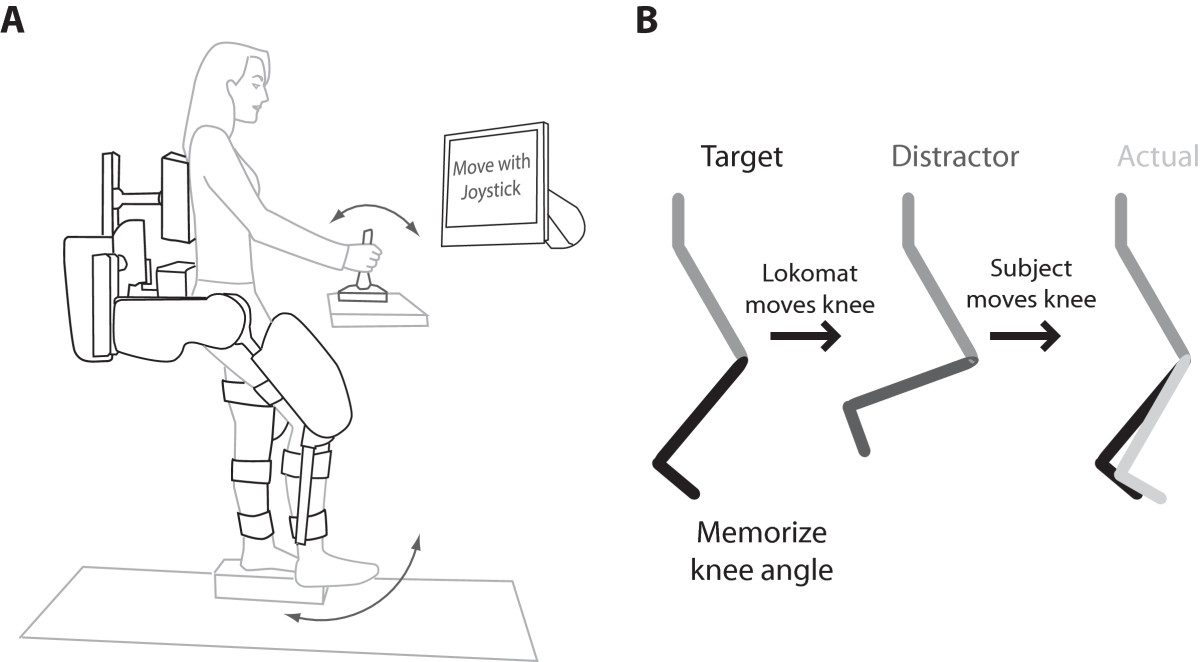
**Experimental set-up and procedures. A**. Subjects were attached to the Lokomat and suspended above the ground using a body weight support system with the ankle fixed in neutral position. All subjects controlled passive leg movements with a joystick. Vision of the legs was obscured with a curtain and only one joint was tested at a time. **B**. Subjects were presented with a target angle, the leg was then moved away from that angle, and the subject used the joystick to place the limb at the remembered target angle. Difference in the target angle and actual angle were recorded as an error.

We used the Lokomat, a robotic lower extremity exoskeleton, and custom software to quantitatively assess lower extremity static joint position sense (Figure 1A). The Lokomat is a computer-controlled motorized gait rehabilitation system consisting of a pair of robotic legs to which the thighs and lower legs are strapped. The thigh and shank segments of the Lokomat only allow movement in the sagittal plane and are moved by linear motors housed within the exoskeletal structure. Encoders within the exoskeleton measure the hip and knee joint angles. The Lokomat was adjusted according to the length and size of the subject's legs. Subjects were secured to the Lokomat by leg cuffs around the mid-thigh, upper shank, and lower shank, as well as a waist belt. Each robotic leg attached to a central horizontal frame that was secured to the subject around the pelvis.

Subjects were suspended in an upright position above the ground using a body weight support system. This helped to ensure that the leg could move freely without touching the treadmill surface. The ankle of the test leg was fixed into a neutral position throughout the experiment with the use of passive foot lifter straps. Foam padding was placed in between the straps and the anterior surface of the limb to decrease any sensory cues from the straps as the leg was moved during testing. Only one leg was tested per subject. In persons with spinal cord injury, we tested the leg with the least amount of spasticity. In able-bodied subjects, we tested the right leg. The foot of the untested leg was placed onto a platform so that the subject could bear some weight on that leg for comfort.

The Lokomat moved the leg into predetermined positions and speeds using custom software. When subjects were asked to move their leg, they all used a joystick controller to change the hip or knee angles. This bypassed restrictions due to variations in the extent of voluntary control over the lower limb between individuals. We set the Lokomat to move the leg at 7° per second, except when using the joystick controller to move the leg, where the speed was 3°or 6° per second, based on the joystick angle. If the subject moved the joystick slightly away from center, the joint moved at 3°/sec, and when pushed further, the leg moved at 6°/sec. We chose to use a different speed when using the joystick controller so that subjects would not use movement time as a cue to the location of the target position. Joint angle data from the encoders were collected using custom software written in LabView (National Instruments, Austin, TX, USA).

### Procedures

Two hip angles (10° extension, 30° flexion) and two knee angles (10° flexion, 50° flexion) were used as target positions for a total of 4 combinations of angles. These angles were chosen because they spanned the range of motion typically used during walking. Each target angle was assessed 5 times. Only one joint was tested at a time, so each combination was done twice, resulting in 40 trials per subject. The order of joint testing was randomized between subjects. Subjects were made aware of which joint was going to be tested for each set of trials. The order of angles tested was randomized within each joint tested. Vision of the legs was obscured with a curtain. Subjects were instructed to keep their leg passive throughout testing. Subjects were given breaks from the body weight support as needed. Blood pressure was measured during the breaks to ensure it stayed near baseline values throughout the study. The assessment was approximately 1.5 hours in duration for each subject, inclusive of all trials and rest breaks.

The hip or knee was moved into a target position and held there for 5 seconds. The subject was asked to memorize the angular position of the joint being tested (the hip or knee). The Lokomat then moved the test joint into another “distractor” position for 5 seconds while the other joint was maintained in the same position. Distractor angles were either 15° or 30° away from the target angle (see Tables 1 & 2). The subject was then asked to bring the test joint back into the memorized target position with the joystick controller. The final joint angle, or “actual” angle, was compared to the target angle and the difference was recorded as an error (Figure 1B).

**Table 1:** **Tasks for hip proprioception assessment**

Task	Hip target angle (°)	Distractor angle (°)	Knee angle (°)
1	30 F	0	10 F
2	30 F	15 F	50 F
3	10 E	5 F	10 F
4	10 E	20 F	50 F

F: Flexion; E: Extension.

**Table 2:** **Tasks for knee proprioception assessment**

Task	Knee target angle (°)	Distractor angle (°)	Hip angle (°)
1	10 F	40 F	30 F
2	10 F	25 F	10 E
3	50 F	35 F	30 F
4	50 F	20 F	10 E

F: Flexion; E: Extension.

These procedures were repeated on a second day at least one week later to assess test-retest reliability.

### Clinical assessment

We also performed a clinical test of proprioception [13] in subjects with SCI, where the leg was moved (either the hip or knee) ~10° by an experimenter from a random starting position, and the subject had to indicate whether the movement was “up” or “down”. This was repeated 10 times at each joint [14], and the number of incorrect responses were compared to the error scores of the robotic assessment. The same experimenter performed the clinical assessment for all subjects.

### EMG

For a subset of subjects (AB: N = 14, SCI: N = 16), EMG data were recorded from the rectus femoris, medial hamstring, medial gastrocnemius and tibialis anterior during the robotic assessment. EMG signals were visually monitored on-line to ensure that the muscles remained quiescent during the tests. If muscle activity was observed (due to voluntary activation or spasticity), the trial was repeated until no muscle activity was observed.

### Data analysis and statistics

All data analysis was performed using IBM SPSS Statistics 21 (New York, NY). At each target angle, we took the absolute values of all the errors. Smaller errors are associated with more accurate joint position sense. Means were reported with ±1 standard error. Significance was evaluated at α = 0.05 for all statistical tests.

#### Discriminative validity

To test if there were differences between groups (SCI vs. AB), target angles, and repetitions, we performed a mixed factorial analysis of variance (ANOVA) (within: 4 target angles x 5 repetitions; between: 2 groups) comparing absolute errors from the first day of testing, separately for the hip and the knee. Post hoc analysis was performed as needed to delineate specific differences between target angles (with a Sidak correction for multiple comparisons). These outcomes provided information on discriminative validity (between groups) of the robotic assessment.

We also performed a mixed factorial ANOVA on the standard deviation of Day 1 error scores (within: 4 target angles; between: 2 groups), separately for the hip and knee. Post hoc analysis was performed as needed to delineate specific differences between target angles (with a Sidak correction for multiple comparisons). Subjects with poorer proprioception and with larger angle errors would also be expected to have greater variability in their responses.

#### Test–retest reliability

In addition to the mixed factorial analysis, we also calculated intraclass correlation coefficients (ICC) [31] and Bland-Altman tests [32] to assess reliability. The intraclass correlation coefficients were calculated on the overall average scores of 20 trials for the hip and knee separately, for able-bodied subjects and subjects with SCI. An ICC value of less than 0.40 indicates poor reproducibility, ICC values in the range 0.40 to 0.75 indicate fair to good reproducibility, and an ICC value of greater than 0.75 shows excellent reproducibility [33].

The Bland-Altman tests include (1) a graphical representation (Bland–Altman plot) of the difference between test measures plotted against the mean of the two measures; (2) calculation of the mean of the difference between test measures and 95% confidence intervals (CI); and (3) a measure of the limits of agreement (LOA) between the two measures, which is defined as d ± 1.96 x SD_diff_, where d is the difference and SD_diff_ is the standard deviation of the differences.

#### Internal consistency

Internal consistency between the different trials was measured using Cronbach’s alpha. We analyzed absolute error scores from the first day of testing, separately for the hip and knee (20 trials at each joint) in both AB and SCI participants. We used this to determine if the number of trials could be reduced for future experiments.

An exploratory factor analysis (principal components method) was also performed to evaluate dimensionality of the assessment. Averaged errors at each target for the SCI data were entered into separate analyses. The criteria for the factors were based on Kaiser stopping criteria, where selected factors had an eigenvalue above 1.0.

#### Convergent validity

Robotic assessment scores were compared to results from the clinical assessment of proprioception. We used Spearman’s rank correlation to compare these results.

## Results

### Participants

Twenty three able-bodied subjects (9 males, 14 females; age = 37.8 ± 14.1 years (mean ± SD)) and 23 subjects with incomplete SCI (19 males, 4 females; age = 40.5 ± 14.0 years, American Spinal Injury Association Impairment Scale (AIS) = B-D, 6.3 ± 5.6 years post-injury) participated in the study. All participants with iSCI were community dwelling but varied greatly with respect to injury and walking ability (Table 3). The AB and SCI groups were not significantly different in terms of age (P = 0.51), but the proportion of males and females were different between groups. Most subjects were able to maintain quiescent muscles throughout the testing period. For some subjects, if any increase in EMG was observed during a trial, subjects were able to reduce their muscle activity when the trial was repeated.

**Table 3:** **Subject demographics and clinical characteristics**

Subject	Sex	Age (yr)	AIS	AIS Level	Time post-injury (yr)	10 MWT (comf) (s)	10 MWT (max) (s)	Assistive device
1	F	55	D	T3	4	25.8	19.8	FC, AFO & FES
2	M	29	B	C6-C7	8	97.7	NT	FWW
3	M	67	C	C4-C5	4	24.8	19.1	FC
4	M	61	B	C5	7	128.3	NT	FWW & Bilateral arm trough
5	F	23	B/C	T12	3	19.1	17.2	FC
6	M	46	C	T12	4	47.8	NT	FWW
7	F	19	B	T12	1	NT	NT	NA
8	M	34	C	C7	18	38.3	27.1	FWW
9	M	47	C	C3-T1	6	11.1	9.9	None
10	M	25	C	T10	2	NT	NT	FC & braces
11	M	65	D	C4-C5	5	20.7	14.5	FC
12	M	41	D	C4-C5	3	7.9	7.3	none
13	M	30	C	T5-T6	10	65.4	55.1	SW & Left AFO
14	M	46	D	C3	2	9.2	7.1	none
15	M	33	B	T4	4	NT	NT	none
16	M	28	C	C5-C6	4	91.7	75.4	FWW
17	F	57	C	T7	23	135.0	86.0	SW
18	M	36	C	C4-5	7	38.1	28.2	FC, swedish cage right knee, right AFO
19	M	49	B	C5	2	NT	NT	NT
20	M	38	B	C7	15	NT	NT	NT
21	M	21	B	C5-C6	4	NT	NT	NT
22	M	42	C	C1-C2	9	16.2	9.6	4 wheeled walker & AFO
23	M	41	C/D	C4-5	1	26.0	15.9	FWW

AIS: American Spinal Injury Association Impairment Scale; 10MWT (comf): 10-meter walk test at comfortable speed, in seconds; 10MWT (max): 10-meter walk test at maximum speed, in seconds; FC: forearm crutches; AFO: ankle-foot orthosis; FES: functional electrical stimulation; FWW: front-wheeled walker; NT: not tested; SW: standard walker; R: right.

### Comparison of absolute errors between groups, target angles, and repetitions

**Figure 2:**
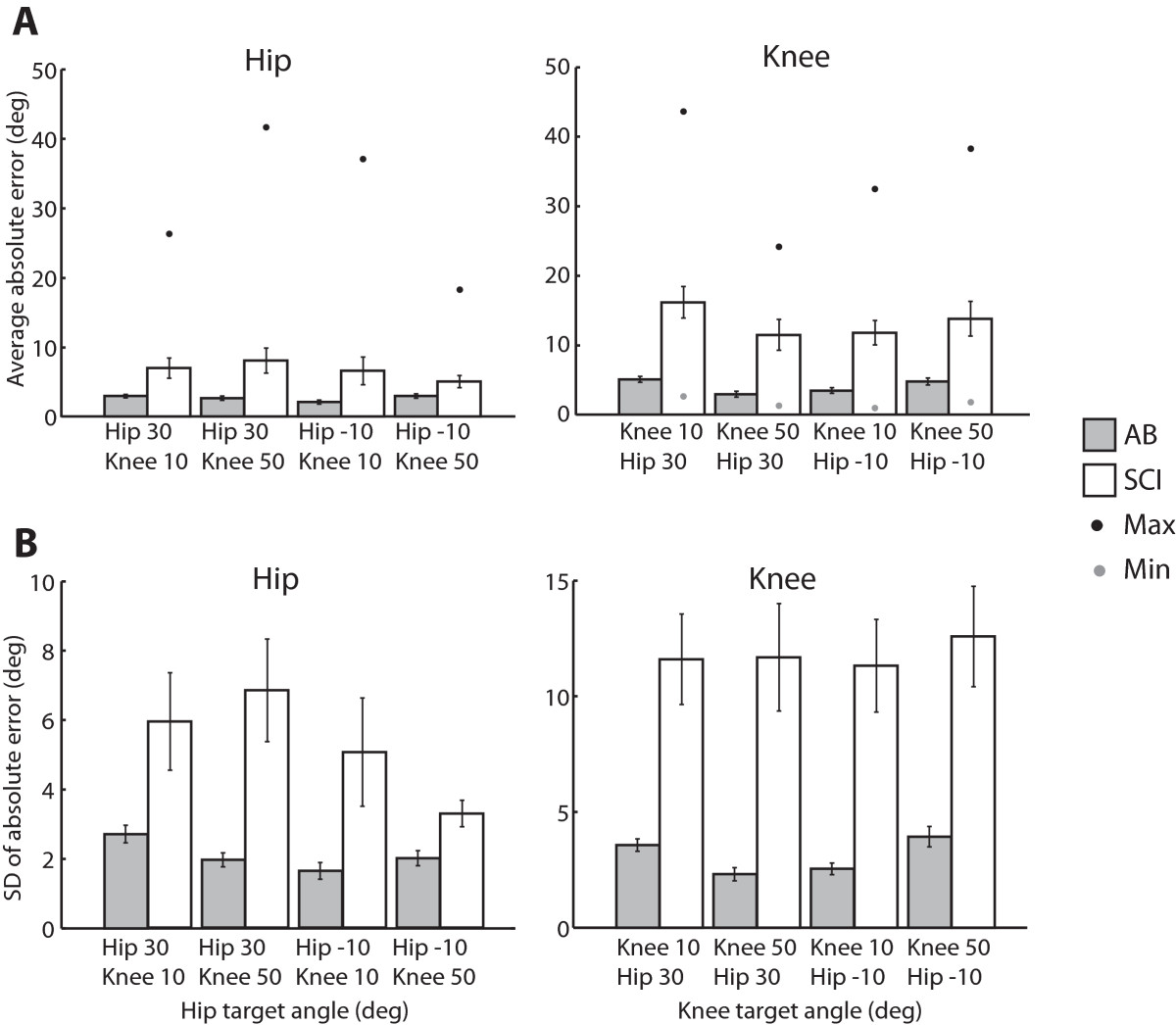
**Absolute angle errors and error variability. A**. Absolute angle errors for the hip and knee joints (mean ± SEM). Angle errors in subjects with SCI were greater than in AB subjects for both joints (P ≤ 0.001, repeated measures ANOVA). There were no significant differences in target angle or repetitions for either group. Angle errors are labeled by knee or hip target angle with respective positions at the other joint. The black and gray dots represent maximum and minimum absolute angle errors, respectively, across subjects for each target. Only one joint was tested at a time. **B**. Standard deviations of angle errors for the hip and knee joints (mean ± SEM). Subjects with SCI had higher standard deviations (were more variable) (P < 0.0001, repeated measures ANOVA) than in AB subjects. There were no significant differences in target angle or repetitions for either group. Angle error standard deviations are labeled by knee or hip target angle with respective positions at the other joint.

Able bodied subjects had an overall average absolute error of 2.63° ± 0.17 (mean ± standard error) at the hip and 4.05° ± 0.28 at the knee, while participants with SCI had an overall average of 6.64° ± 1.18 at the hip and 13.31° ± 1.75 at the knee across 20 trials (5 repetitions of 4 different target angles, Day 1) (Figure 2A). The ANOVA showed that there was a significant difference in error scores between the AB and SCI groups for the hip and knee (P ≤ 0.001 for both joints). At the hip, there were no differences between target angles or repetitions (P = 0.199 and 0.426, respectively, Lower-bound correction due to violation of sphericity). Similarly at the knee, there were no differences between target angles or repetitions (P = 0.067 and 0.392, respectively, Lower-bound correction due to violation of sphericity). There were no interaction effects.

### Comparison of standard deviation of error scores

Subjects with SCI had greater variability (larger standard deviations) in their error scores than able-bodied subjects (hip and knee: P < 0.0001) (Figure 2B). There were no differences in variability between target angles (hip and knee: P > 0.129, Huynh-Feldt correction due to violation of sphericity) or interaction effects (hip and knee: P > 0.207).

### Test-retest reliability

**Figure 3:**
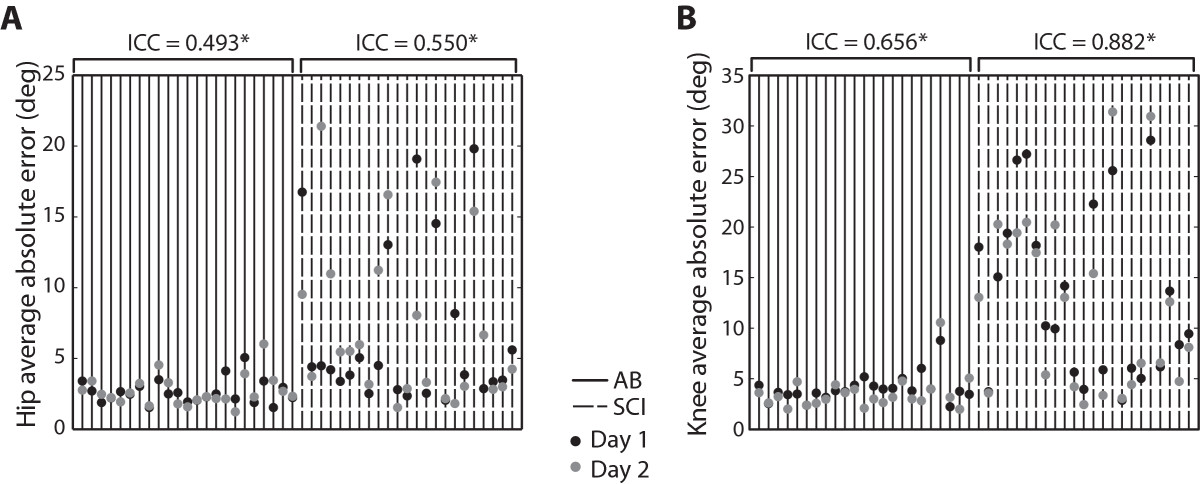
**Test-retest reliability: intraclass correlation coefficients (ICC).** Average absolute angle errors for Day 1 and Day 2 for the **A**. hip joint and **B**. knee joint. ICC analysis showed the test had fair to excellent reproducibility for AB subjects and SCI subjects (all P ≤ 0.001). Significance of the ICC correlation coefficients are indicated by asterisks (P < 0.05).

**Figure 4:**
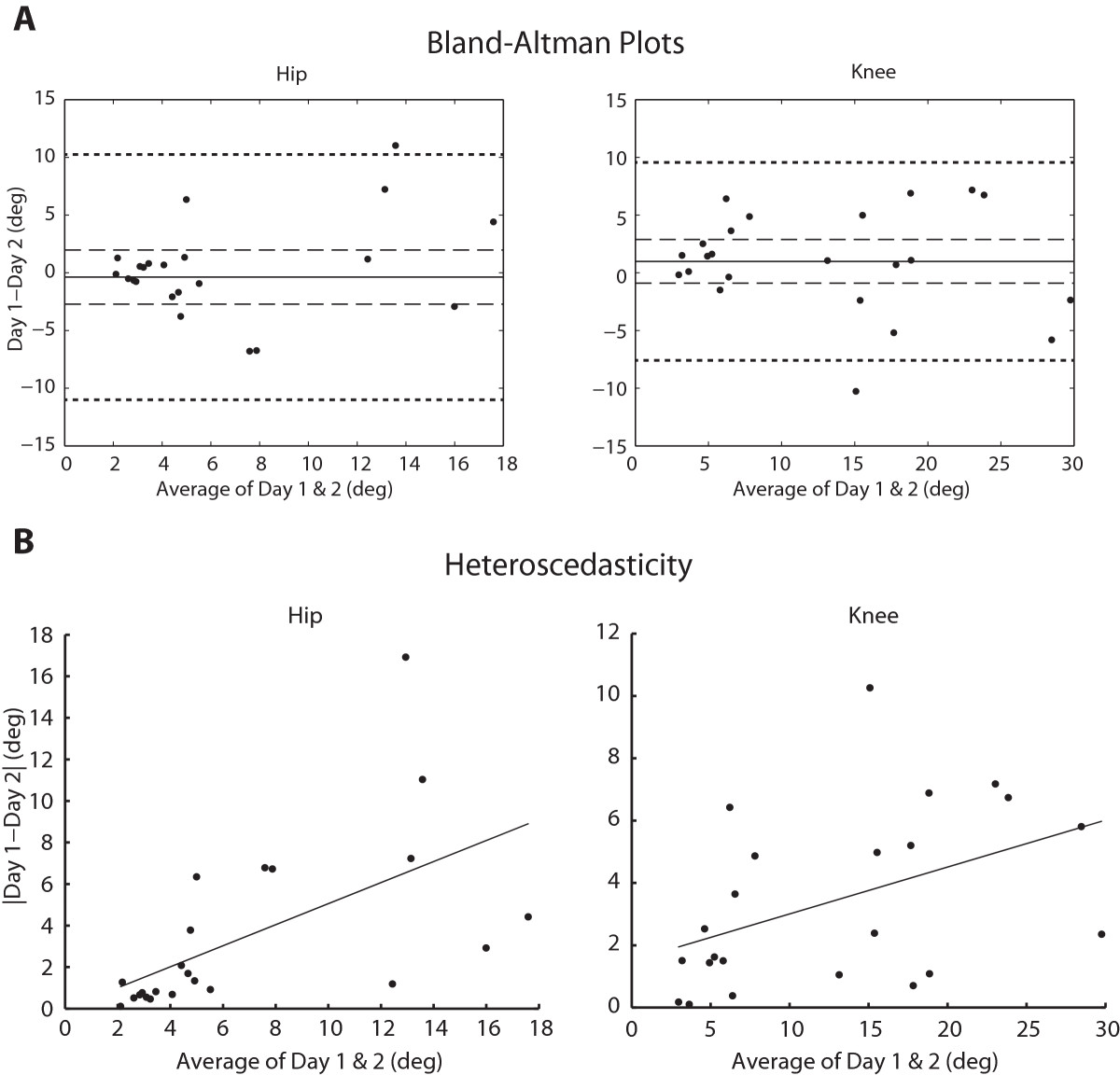
**Bland-Altman plots and heteroscedasticity.** Data points represent data from each participant with SCI. **A**. Bland-Altman plots for agreement of mean absolute angle errors between two separate days of testing for the hip and knee joints. The difference between the two test sessions is plotted versus the mean of the test sessions across days. The 95% confidence intervals of the mean difference between test days are shown as dashed lines. The 95% limits of agreement are shown as dotted lines. **B**. Heteroscedasticity plots for the hip and knee joints in subjects with SCI. There is a positive relationship between the absolute difference and the average of the test session across the two days of testing (hip: P = 0.003; knee: P = 0.031).

The calculated intraclass correlation coefficients show that there was fair to excellent agreement of absolute errors between the 2 days of testing when comparing the overall average scores (Figure 3A & B). In participants with SCI, ICC = 0.55 for the hip assessment and ICC = 0.882 for the knee. In AB participants, ICC = 0.493 for the hip and ICC = 0.656 at the knee (all P ≤ 0.008).The Bland Altman procedures showed that the 95% confidence interval for the mean difference between test sessions (d) included 0 (mean [95% CI] (hip): −0.37 [−2.7, 1.9], mean [95% CI] (knee): 0.98 [−0.90,2.88]), showing there was no systematic change in the errors between days (Figure 4A). The plots do, however, indicate the presence of heteroscedasticity, where the variability of d is unequal across the range of mean errors. In this case, the variability of d is greater at greater error scores. We confirmed the presence of heteroscedasticity by calculating the correlation coefficient between the absolute difference and the average of test sessions and found that it was significant (hip: r = 0.594, P = 0.003; knee: r = 0.451, P = 0.031) (Figure 4B).

### Comparison of robotic assessment to clinical assessment

**Figure 5:**
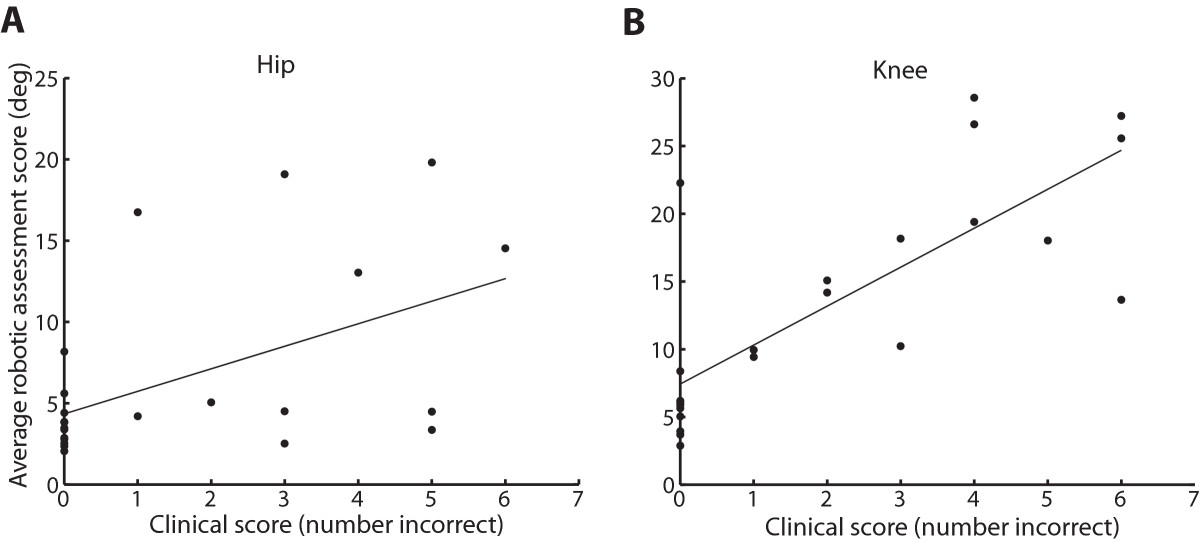
**Comparison of robotic assessment and clinical measures.** Data points represent data from each participant with SCI. Correlation of robot-based assessment to clinical measures at the **A**. hip and **B**. knee. Clinical scores were significantly correlated to robotic assessment scores at both joints (knee: rho = 0.790, P < 0.0001; hip: rho = 0.507, P = 0.013, Spearman’s rank correlation).

In participants with SCI, clinical scores were significantly correlated to robotic assessment scores at both the hip and knee (hip: rho = 0.507, P = 0.013; knee: rho = 0.790, P < 0.0001; Spearman’s rank correlation) (Figure 5A & B).

### Internal consistency

Cronbach’s alpha showed good internal consistency among the different test items in both groups of subjects, and reliability would be minimally affected if an item was deleted from the assessment. In SCI participants, overall α = 0.915 at the hip joint, and if an item was deleted, the range of Cronbach’s alpha was [0.905, 0.919]. At the knee joint, α = 0.868, and if an item was deleted, the range of Cronbach’s alpha was [0.853, 0.874]. In AB participants, overall α = 0.723 at the hip joint, and if an item was deleted, the range was [0.675, 0.756]. At the knee joint, α = 0.764, and if an item was deleted, the range of Cronbach’s alpha was [0.698, 0.777].

### Factor analysis

Factor analysis showed that a clear solution consisting of 1 factor resulted when using principal components, factoring for both the hip and knee in subjects with SCI, based on the scree plots and eigenvalues. For the hip, 43.4% of the variance observed was explained by one factor. For the knee, 44.4% of the variance observed was explained by one factor. This analysis indicated that each target angle contributed to overall proprioception error scores.

## Discussion

We showed that the Lokomat, used with custom software, is a valid and reliable tool to measure proprioceptive sense of the lower extremities in people with incomplete spinal cord injury. This tool was sensitive enough to detect differences in proprioceptive sense between SCI and AB groups, with significantly greater angle errors in the SCI group for both the hip and knee. There was also large variability observed in proprioceptive sense among subjects with spinal cord injury. This was expected, since the degree of injury to the structures that carry conscious proprioceptive sense may vary greatly between individuals. Conscious proprioception is primarily relayed in the dorsal columns [13, 34], and it has been shown that proprioceptive signals are also transmitted to the cerebral cortex via the spinocervical thalamic tract [35, 36]. Proprioceptive sense is derived from sensory information from muscle spindles, golgi tendon organs and skin mechanoreceptors [13].

There are several testing paradigms for assessing proprioceptive sense, but we chose our protocol on what we felt would be most appropriate for the SCI population. Other robotic assessments of proprioception have used contralateral joint matching tasks, particularly in the upper extremities, where movement occurs in the contralateral limb and then is copied in the test limb [21, 37, 38]. The advantage of this method is that there is no reliance on memory to complete the task, and the subject can rely on reference information of the contralateral limb in real time. However, it is suggested that contralateral limb matching tasks may lead to greater matching errors than in ipsilateral tasks because of the required transfer of information between the hemispheres to the respective somatosensory cortices [39]. Even more importantly, in the current study, a joint matching paradigm would not be appropriate because it would be impossible to tell which side the deficit exists (i.e. contralateral matching tasks assume an uninjured side). Investigators have measured joint position sense of hip rotation in older adults [40] and children [41] with cerebral palsy using visual paradigms, but this would be difficult to do in other planes of motion and at other joints in the lower extremities. Another testing paradigm uses voluntary movement of the limb or body segment to achieve the target angles [42]. Because of the high variation in motor ability between subjects with SCI, we chose to keep all movements passive by using a joystick to move the leg to the target angle. Therefore, given the constraints of the limbs and joints tested, our testing protocol is likely the most appropriate for measuring joint position sense of sagittal plane motion in the legs.

### Correlation to clinical test of proprioceptive sense

Clinical measures of proprioception usually involve moving a limb segment in one direction, and then having the patient copy the movement with the opposite limb or verbalize the direction to the clinician [14]. In the present study, the Lokomat based assessment was highly correlated to a clinical measure of proprioception [13]. However, it is likely that the Lokomat based assessment was more a sensitive assessment since we observed a ceiling effect in the clinical score (Figure 5A & B). In subjects where the clinical test showed completely intact proprioception (zero incorrect responses), the Lokomat-based test showed a wide range of angle errors (Figure 5A & B).

The clinical test we used likely contains elements of both static position sense as well as movement sense, but it is one commonly used by clinicians to assess joint position sense [14, 43, 44]. We chose to use this particular clinical assessment because it only involved one limb at a time and the movements were passive. This helped to maintain similar conditions as the Lokomat-based assessment.

### Advantages of robotic assessment

Using the Lokomat with custom software provided a systematic and reliable way to assess proprioceptive sense in persons with SCI. When testing proprioceptive sense, it is very important to be consistent between testing sessions with tested angles, reference angles, time of movement and guessing time [39]. The computer controlled movement helped to keep the testing environment very consistent between trials and testing sessions, as long as the subject was attached to the robot appropriately by the experimenter. Although using a robotic device to test proprioception should be very reliable in theory, error scores may be inconsistent between testing sessions because of intra-subject variability in individuals with spinal cord injury, especially in those that have poor proprioceptive sense. As evidenced by the presence of heteroscedasticity in persons with SCI, the difference in errors between Day 1 and Day 2 of testing tended to be larger when the average error was larger (Figure 4B). This idea is also illustrated in Figure 3A & B, showing that subjects with SCI who had smaller angle errors tended to have more similar scores between Day 1 and Day 2.

### Limitations of robotic assessment

One limitation to using the Lokomat for assessment of proprioception of the legs is that it unable to measure proprioceptive sense at the ankle. Ankle proprioception has been shown to have a major role in maintaining standing balance [45]. In any case, this study shows a means for assessing proprioceptive sense in a quantitative and reliable manner, and this approach could theoretically be implemented in a robotic device developed for testing the ankle [46]. Discomfort in the some of the subjects had occurred during testing due to being suspended in the harness for an extended period of time with relatively little movement of the lower extremities. This was resolved by taking frequent breaks, monitoring signs and symptoms of autonomic dysreflexia (e.g., taking blood pressure intermittently), and encouraging movement during breaks to facilitate blood flow. Reducing the number of total trials would also help to resolve this issue, and is reasonable given the good internal consistency (Cronbach’s alpha of 0.868 for the knee and 0.916 for the hip) of the Lokomat-based assessment.

Our sample population lacked any individuals with lower spinal cord injuries (e.g., cauda equina injuries), therefore the reliability and validity of the Lokomat-based assessment for this group still needs to be evaluated. In addition, it may not be feasible to use this assessment in those with severely limited joint range of motion since movement of the limb segment is needed to test each joint. Individuals with cognitive impairments (e.g., due to traumatic brain injury) may not be able to participate in this assessment due to the attention and memory requirements of the task.

## Conclusions

This work provides evidence that the Lokomat, when used with custom software, can provide a reliable and valid method for quantifying joint position sense. This will be an essential tool when helping to understand the role of proprioception in the recovery of functional tasks such as standing and skilled walking function. Because we found that there were no differences in errors based on the angles tested and good internal consistency between test items, it would be sensible to use only one combination of target and distractor angles for each joint in future protocols. Future studies should also quantitatively measure movement sense. Ultimately these assessments will help aid in the development of therapeutic interventions to improve proprioceptive sense in people with neurological injury, helping to maximize safe participation and quality of life due to improved mobility.

## Authors’ original submitted files for images

Below are the links to the authors’ original submitted files for images. Authors’ original file for figure 1 Authors’ original file for figure 2 Authors’ original file for figure 3 Authors’ original file for figure 4 Authors’ original file for figure 5
