# Supplementary figures and images for: Reliability and validity of using the Lokomat to assess lower limb joint position sense in people with incomplete spinal cord injury
Source: J Neuroeng Rehabil. 2014 Dec 16;11:167. doi: 10.1186/1743-0003-11-167 (PMC4274718; doi:10.1186/1743-0003-11-167)

**A**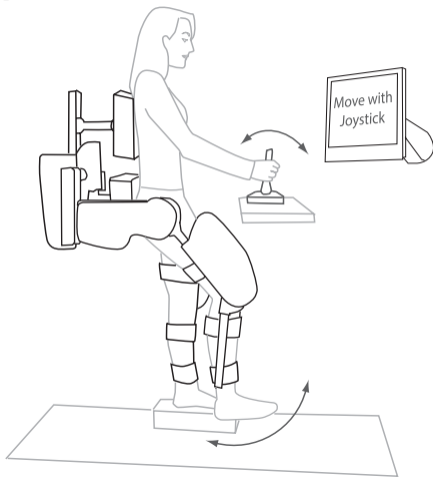**B**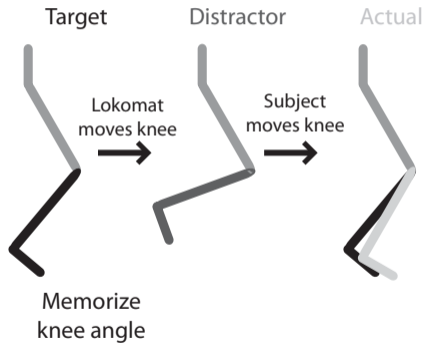

Supplement: Supplementary file 1 — Authors’ original file for figure 1 [file 12984_2014_684_MOESM1_ESM.pdf]

**A**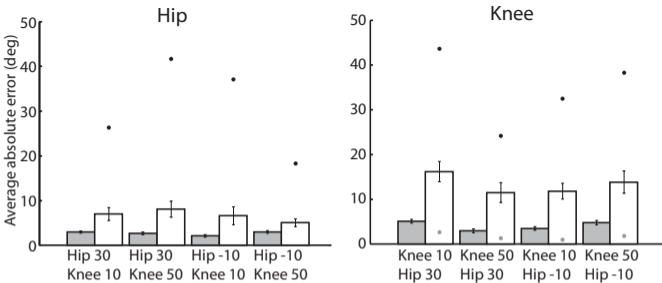**B**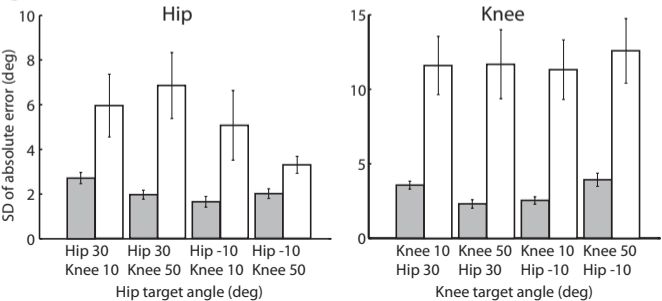

Supplement: Supplementary file 2 — Authors’ original file for figure 2 [file 12984_2014_684_MOESM2_ESM.pdf]

**A**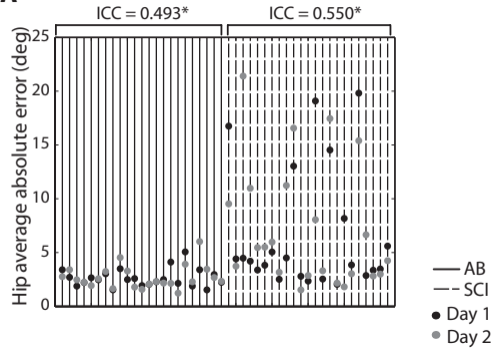**B**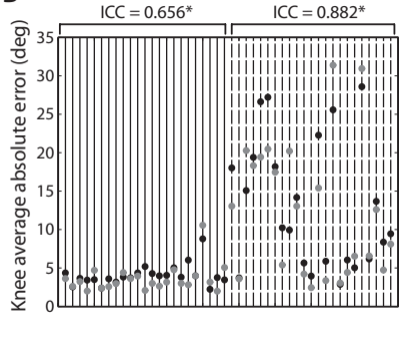

Supplement: Supplementary file 3 — Authors’ original file for figure 3 [file 12984_2014_684_MOESM3_ESM.pdf]

**A**

## Bland-Altman Plots

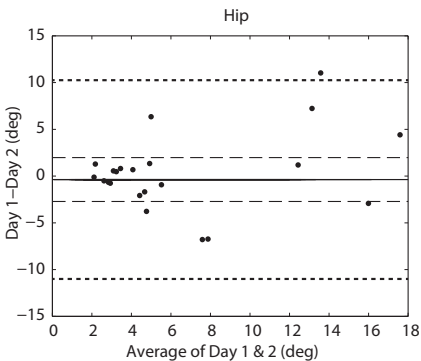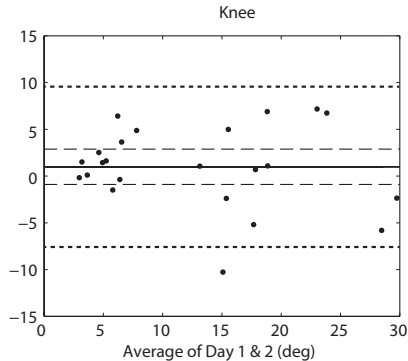**B**

## Heteroscedasticity

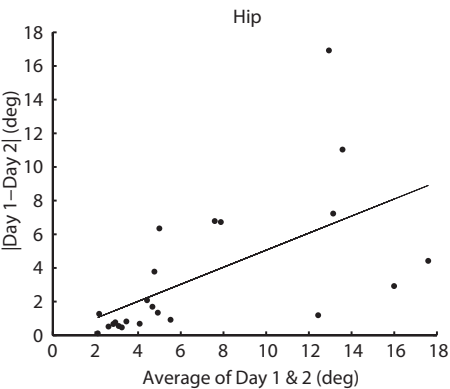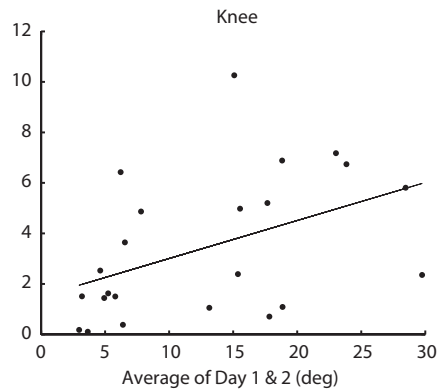

Supplement: Supplementary file 4 — Authors’ original file for figure 4 [file 12984_2014_684_MOESM4_ESM.pdf]

**A**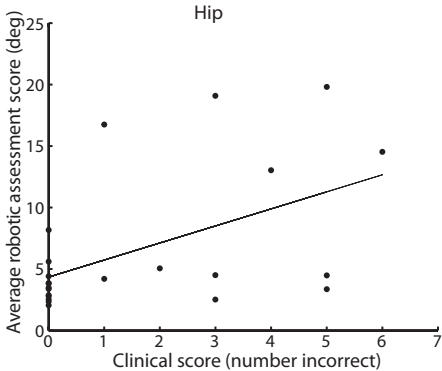**B**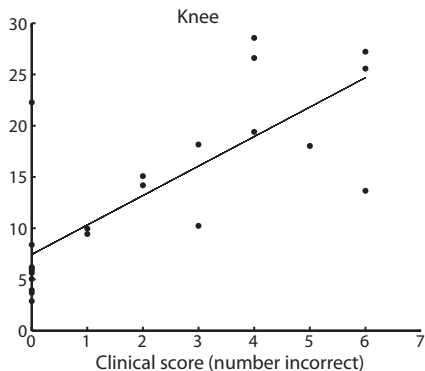

Supplement: Supplementary file 5 — Authors’ original file for figure 5 [file 12984_2014_684_MOESM5_ESM.pdf]
